# Coverage Limitations for Use of Urine Drug Testing in a State Medicaid Program

**DOI:** 10.1001/jamanetworkopen.2026.11711

**Published:** 2026-05-08

**Authors:** Michael A. Incze, Sarah R. Tingley, John Neuhaus, Marcus Bachhuber, S. Amanda Dumas, Sanket S. Dhruva, Rita F. Redberg

**Affiliations:** 1 Division of General Internal Medicine, Department of Medicine, University of Utah School of Medicine, Salt Lake City; 2 University of California, San Francisco School of Medicine, San Francisco; 3 Department of Epidemiology and Biostatistics, University of California, San Francisco, San Francisco; 4 Center for Evidence-based Policy, Oregon Health & Science University, Portland; 5 Center for Healthcare Value and Effectiveness, Section of Community and Population Medicine, School of Medicine, Louisiana State University Health Sciences Center-New Orleans, New Orleans; 6 ForHealth Consulting, UMass Chan Medical School, Worcester, Massachusetts; 7 Section of Cardiology, Department of Medicine, University of California, San Francisco School of Medicine, San Francisco

## Abstract

**Importance:**

Urine drug testing (UDT) is commonly used in substance use disorder (SUD) treatment. However, there is little evidence to guide optimal use of UDT and growing concern that some UDT may represent low-value care.

**Objective:**

To determine whether a statewide policy limiting Medicaid reimbursement for UDT is associated with testing frequency, expenditures, and clinical outcomes.

**Design, Setting, and Participants:**

This serial cross-sectional study was performed among Louisiana Medicaid beneficiaries between July 1, 2017, and February 29, 2020. Data were analyzed from November 1, 2024, to November 30, 2025. For each outcome, best-fit lines for pre-enactment trends were constructed and used to estimate postenactment trends, which were then compared with observed trends. The 3-way interaction of time by intervention period by outcome was analyzed to assess changes for each UDT utilization outcome compared with a matched control procedure (colonoscopy). Concomitant trends in overdose encounters and prescription of medications for opioid use disorder (MOUD) were also assessed.

**Exposure:**

Statewide policy limiting Medicaid reimbursement for UDT enacted in July 2019. Colonoscopy rates were used as a temporal comparison procedure.

**Main Outcomes and Measures:**

Outcomes included rates of monthly UDT (total, presumptive, and definitive) and expenditures per 1000 beneficiaries for 24 months before and 7 months after policy enactment.

**Results:**

The sample included a total of 900 678 unique Medicaid-eligible beneficiaries, 536 841 (59.6%) of whom were female and 606 012 (67.3%) were younger than 40 years. Following policy enactment, the monthly rate of change for total UDT utilization decreased from 0.67 (95% CI, 0.48- 0.85) to −1.03 (95% CI, −1.65 to −0.40) tests per month per 1000 beneficiaries (difference, −1.70 [95% CI, −2.34 to −1.06] tests per month per 1000 beneficiaries); presumptive UDT decreased from 0.42 (95% CI, 0.30-0.53) to −0.63 (95% CI, −0.92 to −0.35) tests per month per 1000 beneficiaries (difference, −1.05 [95% CI, −1.36 to −0.74] tests per month per 1000 beneficiaries); and definitive UDT decreased from 0.25 (95% CI, 0.17-0.34) to −0.39 (95% CI, −0.97 to 0.18) tests per month per 1000 beneficiaries (difference, −0.65 [95% CI, −1.23 to −0.07] tests per month per 1000 beneficiaries). These decreases were all statistically significant compared with colonoscopy (all *P* < .05). UDT expenditures also significantly decreased, totaling an estimated $14.8 million in savings during the 7-month postenactment period. The policy change was not associated with reduced MOUD receipt or increased overdose encounters.

**Conclusion and Relevance:**

In this cross-sectional study, a state policy limiting reimbursement for UDT was associated with significant reductions in UDT utilization and expenditures. Future research and policymaking should investigate ways to optimize UDT for patient health while reducing low-value care.

## Key Points

**Question:**

Is a state Medicaid policy that limited reimbursement for urine drug testing (UDT) associated with UDT utilization, expenditures, and clinical outcomes in opioid use disorder care?

**Findings:**

In this cross-sectional study of 900 678 participants, enactment of UDT coverage policy was associated with immediate and sustained reductions in UDT utilization and expenditures, without any associated change in access to medications for opioid use disorder.

**Meaning:**

These findings suggest that policies that limit reimbursement for UDT may reduce low-value utilization without interrupting access to care.

## Teaser

This cross-sectional study examines the association of changes in coverage of urine drug testing for Medicaid beneficiaries with trends in testing utilization, expenditures, and clinical substance use disorder outcomes.

## Introduction

Preventable deaths attributable to the overdose public health crisis continue to take an immense toll on the lives of US residents.^1^ In 2022 alone, opioid overdose accounted for an estimated 3.1 million years of life lost (38 years per death) in the US.^2^ Despite the burden of death and morbidity attributable to the overdose crisis, fewer than 25% of individuals with substance use disorders (SUDs) receive any treatment.^3,4,5,6^ To address this care gap, training and policies that promote evidence-based use of SUD treatments and assessment tools are necessary. This includes guidance on testing of biological specimens such as urine, hair, saliva, and blood in SUD care. In current clinical practice, urine drug testing (UDT) constitutes the most common form of testing.^7^

UDT is commonly used in SUD treatment to assess for unreported illicit substance use and medication adherence,^8,9^ with US expenditures on UDT eclipsing $8.5 billion in 2017.^10^ However, current guidelines on the optimal use of UDT are based on incomplete evidence and lack detail regarding recommended frequency of testing, specific type of testing (eg, more inexpensive immunoassay-based presumptive tests vs costlier chromatography-based definitive tests), and appropriate response to unexpected results. This has led to widely variable utilization patterns among clinicians.^11^ It also has contributed to inconsistency in how clinicians respond to unexpected test results, with frequent examples of patients being punished or stigmatized rather than having their treatment augmented.^12,13,14^ The high spending on UDT paired with the potential harms and uncertain benefits for patients^14^ have raised concerns of low-value care, defined as a health service for which the potential harms and/or costs outweigh the potential benefits.^15^

One established method of reducing low-value health care services is to enact policies that limit their reimbursement.^16,17,18^ Several states have adopted policies regulating reimbursement for UDT; however, there is substantial heterogeneity between these policies, and to our knowledge no states have published data evaluating their effects on utilization and health outcomes.^19^ Louisiana’s Medicaid policy, enacted in July 2019, limited both the quantity and type of UDT it reimbursed.^20^ Under the policy, Medicaid members could receive a maximum of 24 presumptive UDTs and 18 definitive UDTs per year. Medicaid also ceased reimbursement for definitive UDT of more than 14 drug classes per day in an effort to curb the use of overly expansive (and expensive) testing for substances that may have little clinical relevance to the patient. Prior to policy enactment, there was no formal UDT policy in Louisiana’s Medicaid program. To examine the potential outcomes associated with this policy, we examined trends in UDT utilization, expenditures, and select SUD clinical outcomes.

## Methods

### Study Overview and Inclusion Criteria

We conducted a serial cross-sectional study of all Louisiana Medicaid beneficiaries aged 16 to 64 years from July 1, 2017, to February 29, 2020. Inclusion criteria included at least 1 month of full benefit coverage in the calendar year. Individuals who were covered by multiple entities (eg, dually enrolled in Medicare) were excluded. Our lower limit for age was chosen based on current US Food and Drug Administration approval of buprenorphine to treat opioid use disorder starting at 16 years of age.^21^ We followed the Strengthening the Reporting of Observational Studies in Epidemiology (STROBE) reporting guidelines for reporting this observational study. This research was deemed exempt by the University of California, San Francisco, Institutional Review Board as it was secondary research for which informed consent is not required.

### Outcomes

Our primary outcomes were monthly rates of total, presumptive, and definitive UDT per 1000 Medicaid beneficiaries before and after policy enactment. Secondary outcomes included (1) monthly expenditures on UDT per 1000 beneficiaries, (2) monthly prescriptions filled for medications to treat opioid use disorder (MOUD; ie, US Food and Drug Administration–approved formulations of buprenorphine and extended-release naltrexone), and (3) monthly rates of health care encounters (including inpatient, outpatient, and emergency department settings) for opioid overdose before and after policy enactment. MOUD receipt and overdose encounters were included to determine whether changes in UDT utilization were associated with collateral changes in markers of SUD treatment provision and/or opioid-related harms. We compared trends before and after the policy enactment for each utilization outcome with trends in colonoscopy, a matched control procedure unaffected by specific concurrent policy changes, to contextualize our results within broader trends of health care utilization during the study period.

### Coincident Policy Changes

Working with Louisiana Medicaid administrative staff and leadership, we sought to identify coincident state policy changes that may have affected our results. In February 2019, the Louisiana Department of Health began conducting quarterly income eligibility checks among Medicaid members.^22,23^ Members who were identified as possibly having incomes that were too high to qualify for Medicaid coverage were mailed a letter requesting income verification. Children, pregnant individuals, and members who were in active plan renewal were excluded. Failure to respond to this letter within 10 days led to a termination of coverage. During the first 3 months of the program, 40 000 income verification requests were issued, and more than 30 000 members experienced coverage interruptions.^22^

### Data Collection

Medicaid fee-for-service claims and managed care encounter data were obtained and analyzed for the state of Louisiana between July 2017 and February 2020. Our date range was chosen in part due to the onset of the COVID-19 pandemic, which caused meaningful disruption of in-person SUD treatment and assessment in the Spring of 2020.

Data on age, sex, and race were collected from Louisiana Medicaid claims. Race data were categorized as self-identified American Indian or Alaska Native, Asian, Black, Native Hawaiian or Other Pacific Islander, White, or other or unknown; these data were collected to characterize our sample and examine trends UDT among different subgroups after policy enactment. *Current Procedural Terminology* (*CPT*) codes were used to identify monthly counts and expenditures on presumptive and definitive UDT (eTable 1 in Supplement 1). Of note, after July 2019, billing for UDT switched from *CPT* coding (where each component of the UDT could be coded as a separate test) to bundled G codes that applied a single code to every component in a single UDT. For example, prior to July 2019, a UDT that tested for 5 substances could be associated with 5 separate *CPT* codes (generating 5 separate claim lines), whereas after July 2019 the same test would be coded with a single G code. To standardize our comparison, in the pre-enactment period we considered all UDT *CPT* codes billed for a single beneficiary by a single clinician on a single date of service to be a single test.

National Drug Codes were used to identify monthly beneficiaries receiving prescriptions for MOUD. Of note, we could not examine methadone or other MOUDs administered within federally regulated opioid treatment programs because those services were not covered by Louisiana’s Medicaid program until January 20, 2020.^24^ Codes from *International and Statistical Classification of Disease, Tenth Revision*, were used to identify opioid overdose encounters across inpatient, outpatient, and emergency department settings (eTable 2 in Supplement 1).

### Statistical Analysis

Data were analyzed between November 1, 2024, and November 30, 2025. We performed an interrupted time series analysis comparing expected vs observed trajectories for each outcome following policy enactment in July 2019. We constructed best-fit lines for each outcome during the 24 months preceding policy enactment. We used these lines to estimate postenactment trends and then compared these estimations with observed trends for each outcome during a 7-month period following policy enactment. The interrupted time series analysis assessed the direction and magnitude of change between estimated and observed trends after policy enactment.

For 1 outcome (overdose encounters), our initial interrupted time series analysis produced results that did not appear to account for evolving changes in overdose trends occurring during the period immediately preceding policy enactment. Thus, we performed 2 additional analyses for months 0 to 24 (point of interruption, month 16) and months 16 to 32 (point of interruption, month 24 [policy enactment]). These analyses were intended to more closely investigate how overdose trends immediately preceding policy enactment were associated with postenactment trends.

Finally, we compared trends before and after policy enactment for utilization and expenditure outcomes with colonoscopy, a matched control procedure that was not specifically affected by concurrent policy changes. This analysis examined the 3-way interaction of time by intervention period (ie, before and after policy enactment) by outcome vs the control procedure. Two-tailed *P* < .05 was considered statistically significant. All analyses were performed using Stata, version 16.1 (StataCorp LLC).

## Results

Our sample included a total of 900 678 unique Medicaid-eligible beneficiaries aged 16 to 64 years from July 2017 through February 2020. Among our sample, 536 841 (59.6%) beneficiaries were female and 363 837 (40.4%) were male. In terms of self-reported race, 6945 beneficiaries (0.8%) were American Indian or Alaska Native; 13 521 (1.5%), Asian; 417 849 (46.4%), Black; 3194 (0.4%), Native Hawaiian or Other Pacific Islander; 374 032 (41.5%), White; and 85 137 (9.5%), other or unknown race (Table 1).

**Table 1.** Demographic Characteristics of Included Louisiana Medicaid Beneficiaries

Characteristic	No. (%) of beneficiaries (N = 900 678)
Sex	
Male	363 837 (40.4)
Female	536 841 (59.6)
Age category, y	
16-24	264 030 (29.3)
25-39	341 982 (38.0)
40-64	294 666 (32.7)
Race^a^	
American Indian or Alaska Native	6945 (0.8)
Asian	13 521 (1.5)
Black	417 849 (46.4)
Hawaiian Native or Other Pacific Islander	3194 (0.4)
White	374 032 (41.5)
Other or unknown^b^	85 137 (9.5)

^a^ Collected from self-identification from Louisiana Medicaid claims.

^b^ Category was not disaggregated in our dataset.

**Figure 1.**
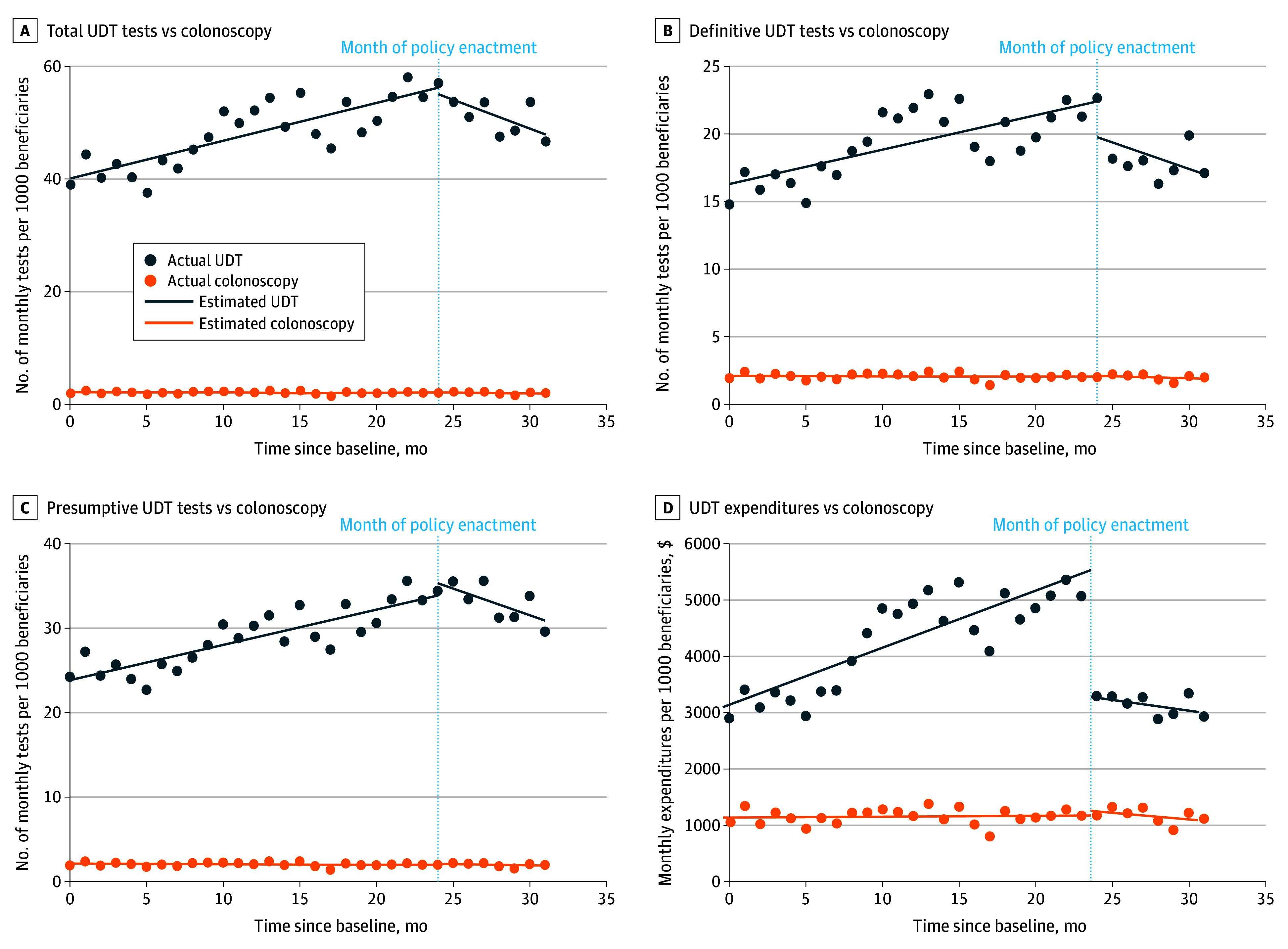
Line Graph of Plotted Trends in Urine Drug Testing (UDT) Utilization and Expenditures Before and After Policy Enactment Compared With Colonoscopy

Overall, there were significant decreases in monthly utilization of total UDT, definitive UDT, and presumptive UDT after policy enactment (Figure 1). These reductions in utilization were statistically significant compared with concomitant trends in colonoscopy. There was also a statistically significant decrease in monthly expenditures on UDT (eTable 3 in Supplement 1).

### Number and Type of UDT

In the 24 months preceding policy enactment, the monthly rate of total UDT increased by 0.67 (95% CI, 0.48-0.85) tests per month per 1000 beneficiaries. In the 7 months following policy enactment, the rate decreased by −1.03 (95% CI, −1.65 to −0.40) tests per month per 1000 beneficiaries. This corresponds to a significant decrease in monthly UDT utilization following policy enactment (difference, −1.70; [95% CI, −2.34 to −1.06]) (Table 2). Three-way analysis demonstrated a significantly greater reduction in rates of total monthly UDT utilization compared with colonoscopy after policy enactment (−1.67 [95% CI, −2.31 to −1.03] tests per month per 1000 beneficiaries).

**Table 2.** Changes in Monthly UDT Utilization and Expenditure Trends Per 1000 Medicaid Beneficiaries Before and After Policy Enactment

UDT category	Monthly utilization rate (95% CI) per 1000 beneficiaries		Difference in policy rates (95% CI) per 1000 beneficiaries	Three-way interaction comparing UDT trends with colonoscopy per 1000 beneficiaries^a^
	Before policy enactment	After policy enactment		
Total UDT counts	0.67 (0.48 to 0.85)	−1.03 (−1.65 to −0.40)	−1.70 (−2.34 to −1.06)	−1.67 (−2.31 to −1.03)
Presumptive UDT counts	0.42 (0.30 to 0.53)	−0.63 (−0.92 to −0.35)	−1.05 (−1.36 to −0.74)	−1.02 (−1.33 to −0.71)
Definitive UDT counts	0.25 (0.17 to 0.34)	−0.39 (−0.97 to 0.18)	−0.65 (−1.23 to −0.07)	−0.62 (−1.20 to −0.04)
Total UDT expenditures, $	101.56 (80.86 to 122.05)	−38.22 (−69.70 to −6.74)	−139.68 (−176.98 to −102.37)	−113.93 (−159.01 to −68.86)
Colonoscopy counts (control)	−0.003 (−0.013 to 0.006)	−0.033 (−0.079 to 0.011)	−0.03 (−0.08 to 0.02)	NA
Colonoscopy expenditures (control), $	1.29 (−4.83 to 7.41)	−24.46 (−49.00 to 0.09)	−25.74 (−51.05 to −0.44)	NA

Abbreviations: NA, not applicable; UDT, urine drug testing.

^a^ Calculated as time × before and after policy change × UDT vs colonoscopy.

Rates of presumptive UDT decreased from 0.42 (95% CI, 0.30-0.53) tests per month per 1000 beneficiaries before policy enactment to −0.63 (95% CI, −0.92 to −0.35) tests per month per 1000 beneficiaries after enactment (difference, −1.05 [95% CI, −1.36 to −0.74] tests per month per 1000 beneficiaries); definitive UDT decreased from 0.25 (95% CI, 0.17-0.34) tests per month per 1000 beneficiaries before enactment to −0.39 (95% CI, −0.97 to 0.18) tests per month per 1000 beneficiaries after enactment (difference, −0.65 [95% CI, −1.23 to −0.07] tests per month per 1000 beneficiaries) (Table 2). Three-way analysis demonstrated significantly greater reductions in monthly presumptive (−1.02 [95% CI, −1.33 to −0.71] tests per month per 1000 beneficiaries) and definitive (−0.62 [95% CI, −1.20 to −0.04] tests per month per 1000 beneficiaries) UDT utilization compared with colonoscopy.

### Expenditures

In the 24 months preceding policy enactment, total monthly expenditures for UDT increased by $101.56 (95% CI, $80.86-$122.05) per month per 1000 beneficiaries. In the 7 months following policy enactment, the rate decreased by −$38.22 (95% CI, −$69.70 to −$6.74) per month. There was a significant decrease in the rate of UDT expenditures immediately following policy enactment (difference, −$139.68 [95% CI, −$176.98 to −$102.37] per month per 1000 beneficiaries) (Table 2). Three-way analysis demonstrated a significantly greater reduction in monthly total UDT expenditures compared with expenditures for colonoscopy (−$113.93 [95% CI, −$159.01 to −$68.86] per month per 1000 beneficiaries). Total estimated savings during the 7-month postenactment period, calculated by comparing observed monthly expenditures with expected expenditures based on pre-enactment trends, were estimated to be $14.8 million.

### MOUD Receipt

**Figure 2.**
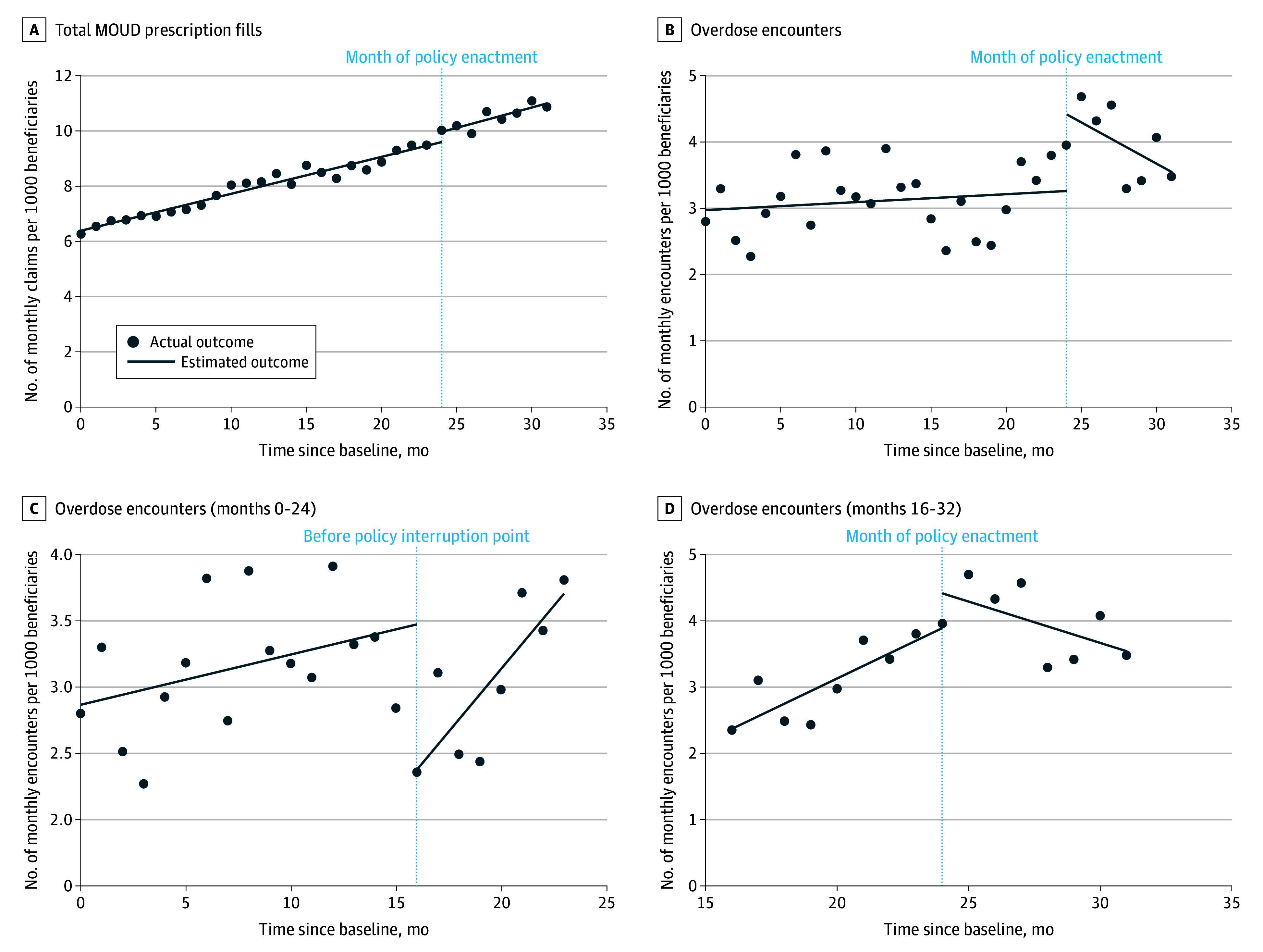
Line Graph of Plotted Trends in Medication for Opioid Use Disorder (MOUD) Prescription Fills and Overdose Encounter Rates Before and After Policy Enactment Overdose encounter trends are presented using 3 separate regression models: the entire study period, with the interruption point at the month of policy enactment; the pre-enactment period only, with the interruption point at month 16; and the 8-month periods preceding and following policy enactment, with the interruption point at the month of policy enactment. All rates are per 1000 Medicaid beneficiaries.

Table 3 presents trends in MOUD receipt. In the 24 months preceding policy enactment, the monthly rate of MOUD prescriptions filled increased by 0.13 (95% CI, 0.12-0.14) prescription fills per month per 1000 beneficiaries. In the 7 months following policy enactment, the rate increased by 0.15 (95% CI, 0.11-0.18) prescription fills per month per 1000 beneficiaries. There was no significant difference in rates of MOUD receipt following policy enactment (0.01 [95% CI, −0.02 to 0.05] prescription fills per month per 1000 beneficiaries) (Figure 2A).

**Table 3.** Changes in Monthly MOUD Receipt and Overdose Encounter Trends Per 1000 Medicaid Beneficiaries Before and After Policy Enactment

Outcome	Monthly rate (95% CI) per beneficiaries		Difference in policy rates per 1000 beneficiaries
	Before policy enactment	After policy enactment	
MOUD receipt	0.13 (0.12 to 0.14)	0.15 (0.11 to 0.18)	0.01 (−0.02 to 0.05)
Overdose encounters	0.01 (−0.02 to 0.04)	−0.12 (−0.22 to −0.03)	−0.14 (−0.23 to −0.04)

Abbreviation: MOUD, medications for opioid use disorder.

### Overdose Encounters

Trends in overdose encounters are shown in Table 3. In the 24 months preceding policy enactment, the monthly rate of overdose encounters increased by 0.01 (95% CI, −0.02 to 0.04) per 1000 beneficiaries. In the 7 months following policy enactment, the rate decreased by −0.12 (95% CI, −0.22 to −0.03) per 1000 beneficiaries (Figure 2B). This represents a significant decrease in monthly overdose encounter trends after policy enactment (difference, −0.14 [95% CI, −0.23 to −0.04] per 1000 encounters). As shown in Figure 2B, it initially appeared that there was an abrupt increase in total overdose encounters in the month following policy enactment. However, additional analyses demonstrated that overdose encounter rates significantly increased starting 8 months before policy enactment compared with the antecedent 15 months of the pre-enactment period (difference, 0.15 [95% CI, 0.005-0.30] encounters per month per 1000 beneficiaries) (Figure 2C). Extending this trend to the postenactment period, the observed rate of overdose encounters in the month following policy enactment was not significantly different than estimated (difference, 0.52 [95% CI, −0.15 to 1.19] encounters per month per 1000 beneficiaries). Adding the additional interruption point at month 16 reduced the residual mean square error by more than 20% (Figure 2C), indicating a substantial improvement in model fit and suggesting that there was not a significant increase in overdose encounters in the month following policy enactment (Figure 2D).

## Discussion

Despite the ubiquity of UDT in clinical practice, its lack of supporting evidence, high costs, variable implementation, and potential harms for patients indicate that at least some UDT may represent low-value care. To curb potential low-value use of UDT, Louisiana’s Medicaid program implemented a policy in July 2019 that limited the number and types of UDT it would reimburse. Our results demonstrate that this policy was associated with significant decreases in UDT utilization and expenditures in the postenactment period, totaling an estimated $14.8 million in savings during just 7 months of observation. The 3-way interaction analyses demonstrated significantly greater reductions in UDT utilization and spending than for colonoscopy, a control procedure that to our knowledge was not affected by specific coverage policy changes during the observation period. Taken together, these results suggest that policies limiting reimbursement for UDT can be an effective way to reduce utilization and expenditures.

Our analysis looked at 2 potential collateral harms of placing limits on UDT reimbursement: MOUD receipt and overdose encounters. These outcomes were included to determine whether restricting UDT may have affected access to SUD treatment. For example, restricting use of UDT might discourage clinicians from prescribing MOUD and/or hinder adequate assessment of substance use patterns that could ultimately lead to undertreatment and increased overdose risk. Conversely, limiting UDT might preserve SUD treatment access and lower overdose risk by reducing stigma and confrontation about unexpected UDT results in clinical care.

For MOUD, our analysis showed an uninterrupted trend toward increasing MOUD prescriptions before and after policy enactment, suggesting that restricting UDT was not significantly associated with a change in MOUD prescribing among Medicaid members. For overdose encounters, we found that while rates did not significantly increase after policy enactment, it initially appeared that there was an increase in overdose encounters during the month the policy went into effect (Figure 2B). However, a more careful look at data demonstrates that overdose encounter rates began significantly increasing approximately 8 months prior to policy enactment (Figure 2C), with overdose encounters immediately following policy enactment as estimated based on the preceding 8 months (Figure 2D). This contradicts the notion that overdose rates may have increased as a result of the UDT policy. Rather, trends in overdose encounters are likely influenced by numerous complex factors such as the composition of the local drug supply, treatment access, and naloxone availability.^25,26,27,28,29^ Further research is needed to investigate the effects of UDT policy on overdose-related outcomes.

Our work should be interpreted within the context of contemporary policy and practice trends related to UDT. As of September 2025, at least 25 states, in addition to large national insurance providers such as UnitedHealth Group, have implemented policies related to UDT reimbursement.^19^ Utah’s policy, which was enacted in 2022 and imposed similar coverage limits to the policy evaluated in this study, has produced an estimated 17% year-by-year reduction in utilization, although expenditure and clinical outcomes data are not yet available.^30^ Other states such as Michigan have enacted novel policies supporting expanded use of UDT within the context of evidence-based treatment programs such as contingency management,^31,32,33,34,35,36,37,38^ where participants are provided financial incentives for consecutive UDT demonstrating abstinence as a part of an overarching treatment strategy.^39^ Professional societies have also telegraphed evolving views on UDT. For example, the American Society of Addiction Medicine recently recommended that patients retain the right to refuse UDT without facing termination of treatment and have generally called for more judicious and transparent testing approaches.^40,41^ However, there is a dearth of evidence to inform this shifting policy landscape. Our study advances the field of high-value SUD care by demonstrating that state-level policymaking may be an effective way to reduce overutilization of UDT, leading to significantly reduced expenditures in the Medicaid program without an association with worse clinical outcomes.

Our study has important implications for future research, guideline development, and policymaking related to UDT. First, further research is needed to better characterize the association between policies that restrict UDT and clinical outcomes, in addition to delineating the appropriate use of UDT to maximize clinical benefit for patients with SUD. Second, future studies should examine the effects of coverage policies on outliers in testing frequency to inform targeted efforts to reduce low-value care. Finally, until there is more clear evidence showing benefit from routine UDT, guidelines and policy should focus on promoting judicious and parsimonious testing. These policies should also emphasize noncoercive shared decision-making with patients around UDT and the creation of clinical protocols ensuring accurate test interpretation and strengthening—rather than terminating—care in response to unexpected results.

### Limitations

Our study has important limitations. First, we analyzed claims and encounter data, which precluded an investigation into how changes in UDT affected clinical outcomes such as overdose and treatment access at the individual level. Our data also did not allow us to examine trends in UDT for individual clinics or clinicians. Second, our use of claims data and *CPT* codes may have missed UDT that was not billed to insurance (eg, cash pay for patients) or misclassified some MOUD that was prescribed for other indications,^42^ although we suspect that this represents a small fraction of our sample.^43^ Third, our data did not include methadone or other MOUD administered at federally regulated opioid treatment programs, because those services were not covered in Louisiana’s Medicaid program until near the end of the study period. Fourth, the coincident implementation of Medicaid income eligibility checks may have disproportionately caused coverage interruptions among individuals with SUD,^44^ thus affecting use of UDT and insurance claims independent of the UDT policy. Fifth, we were able to observe postenactment trends for only 7 months before the COVID-19 pandemic drastically interrupted in-person care access. Last, trends in other policy and health care utilization at the state, administrative (eg, Medicaid managed care organization), and organizational (eg, individual clinics) levels may have impacted UDT utilization and expenditures in ways that were not accounted for in our study design.

## Conclusions

While UDT represents a potentially useful tool in SUD assessment, there are concerns that current utilization patterns may often represent low-value care. In this cross-sectional study, a Louisiana Medicaid policy that limited reimbursement for UDT was associated with a subsequent reduction in UDT utilization and expenditures without an increase in observed collateral harms. Our study highlights the importance of reconsidering current UDT practices and reimbursement structures nationwide.

## Notes

Supplement 1. **eTable 1.** *Current Procedural Terminology* (*CPT*) Codes Used to Identify Monthly Counts and Expenditures on Presumptive and Confirmatory UDT **eTable 2.** *ICD-10* Codes Used to Identify Opioid Overdose Encounters **eTable 3.** Regression Results

Supplement 2.
Data Sharing Statement
