# Supplementary material for: Coverage Limitations for Use of Urine Drug Testing in a State Medicaid Program
Source: JAMA Netw Open. 2026 May 8;9(5):e2611711. doi: 10.1001/jamanetworkopen.2026.11711 (PMC13156783; doi:10.1001/jamanetworkopen.2026.11711)
Supplement: Supplement 1. — eTable 1. Current Procedural Terminology (CPT) Codes Used to Identify Monthly Counts and Expenditures on Presumptive and Confirmatory UDT eTable 2. ICD-10 Codes Used to Identify Opioid Overdose Encounters eTable 3. Regression Results [file jamanetwopen-e2611711-s001.pdf]

Supplemental Online Content

Incze MA, Tingley SR, Neuhaus J, et al. Coverage limitations for use of urine drug testing in a state Medicaid program. *JAMA Netw Open.* 2026;9(5):e2611711. doi:10.1001/jamanetworkopen.2026.11711

eTable 1. *Current Procedural Terminology* (*CPT*) Codes Used to Identify Monthly Counts and Expenditures on Presumptive and Confirmatory UDT

eTable 2. *ICD-10* Codes Used to Identify Opioid Overdose Encounters

eTable 3. Regression Results

This supplemental material has been provided by the authors to give readers additional information about their work.

© 2026 Incze MA et al. *JAMA Network Open*.

eTable 1. *Current Procedural Terminology* (*CPT*) Codes Used to Identify Monthly Counts and Expenditures on Presumptive and Confirmatory UDT

Category	Included Codes
**Presumptive Testing**	80305, 80306, 80307
**Definitive Testing**	80320-80373, 80375, 80376, 80377, G0480, G0481, G0482, G0483, G0659

eTable 2. *ICD-10* Codes Used to Identify Opioid Overdose Encounters

Code	Type	Description
96500	ICD-9	Poisoning by opium (alkaloids), unspecified
96501	ICD-9	Poisoning by heroin
96502	ICD-9	Poisoning by methadone
96509	ICD-9	Poisoning by other opiates and related narcotics
E8500	ICD-9	Accidental poisoning by heroin
E8501	ICD-9	Accidental poisoning by methadone
E8502	ICD-9	Accidental poisoning by other opiates and related narcotics
T400X1A	ICD-10	Poisoning by opium, accidental (unintentional), initial encounter
T400X1D	ICD-10	Poisoning by opium, accidental (unintentional), subsequent encounter
T400X1S	ICD-10	Poisoning by opium, accidental (unintentional), sequela
T400X2A	ICD-10	Poisoning by opium, intentional self-harm, initial encounter
T400X2D	ICD-10	Poisoning by opium, intentional self-harm, subsequent encounter
T400X2S	ICD-10	Poisoning by opium, intentional self-harm, sequela
T400X3A	ICD-10	Poisoning by opium, assault, initial encounter
T400X3D	ICD-10	Poisoning by opium, assault, subsequent encounter
T400X3S	ICD-10	Poisoning by opium, assault, sequela
T400X4A	ICD-10	Poisoning by opium, undetermined, initial encounter
T400X4D	ICD-10	Poisoning by opium, undetermined, subsequent encounter
T400X4S	ICD-10	Poisoning by opium, undetermined, sequela
T401X1A	ICD-10	Poisoning by heroin, accidental (unintentional), initial encounter
T401X1D	ICD-10	Poisoning by heroin, accidental (unintentional), subsequent encounter
T401X1S	ICD-10	Poisoning by heroin, accidental (unintentional), sequela
T401X2A	ICD-10	Poisoning by heroin, intentional self-harm, initial encounter
T401X2D	ICD-10	Poisoning by heroin, intentional self-harm, subsequent encounter
T401X2S	ICD-10	Poisoning by heroin, intentional self-harm, sequela
T401X3A	ICD-10	Poisoning by heroin, assault, initial encounter
T401X3D	ICD-10	Poisoning by heroin, assault, subsequent encounter
T401X3S	ICD-10	Poisoning by heroin, assault, sequela
T401X4A	ICD-10	Poisoning by heroin, undetermined, initial encounter
T401X4D	ICD-10	Poisoning by heroin, undetermined, subsequent encounter

© 2026 Incze MA et al. *JAMA Network Open*.

T401X4S	ICD-10	Poisoning by heroin, undetermined, sequela
T402X1A	ICD-10	Poisoning by other opioids, accidental (unintentional), initial encounter
T402X1D	ICD-10	Poisoning by other opioids, accidental (unintentional), subsequent encounter
T402X1S	ICD-10	Poisoning by other opioids, accidental (unintentional), sequela
T402X2A	ICD-10	Poisoning by other opioids, intentional self-harm, initial encounter
T402X2D	ICD-10	Poisoning by other opioids, intentional self-harm, subsequent encounter
T402X2S	ICD-10	Poisoning by other opioids, intentional self-harm, sequela
T402X3A	ICD-10	Poisoning by other opioids, assault, initial encounter
T402X3D	ICD-10	Poisoning by other opioids, assault, subsequent encounter
T402X3S	ICD-10	Poisoning by other opioids, assault, sequela
T402X4A	ICD-10	Poisoning by other opioids, undetermined, initial encounter
T402X4D	ICD-10	Poisoning by other opioids, undetermined, subsequent encounter
T402X4S	ICD-10	Poisoning by other opioids, undetermined, sequela
T403X1A	ICD-10	Poisoning by methadone, accidental (unintentional), initial encounter
T403X1D	ICD-10	Poisoning by methadone, accidental (unintentional), subsequent encounter
T403X1S	ICD-10	Poisoning by methadone, accidental (unintentional), sequela
T403X2A	ICD-10	Poisoning by methadone, intentional self-harm, initial encounter
T403X2D	ICD-10	Poisoning by methadone, intentional self-harm, subsequent encounter
T403X2S	ICD-10	Poisoning by methadone, intentional self-harm, sequela
T403X3A	ICD-10	Poisoning by methadone, assault, initial encounter
T403X3D	ICD-10	Poisoning by methadone, assault, subsequent encounter
T403X3S	ICD-10	Poisoning by methadone, assault, sequela
T403X4A	ICD-10	Poisoning by methadone, undetermined, initial encounter
T403X4D	ICD-10	Poisoning by methadone, undetermined, subsequent encounter
T403X4S	ICD-10	Poisoning by methadone, undetermined, sequela
T404X1A	ICD-10	Poisoning by other synthetic narcotics, accidental (unintentional), initial encounter
T404X1D	ICD-10	Poisoning by other synthetic narcotics, accidental (unintentional), subsequent encounter
T404X1S	ICD-10	Poisoning by other synthetic narcotics, accidental (unintentional), sequela
T404X2A	ICD-10	Poisoning by other synthetic narcotics, intentional self-harm, initial encounter
T404X2D	ICD-10	Poisoning by other synthetic narcotics, intentional self-harm, subsequent encounter
T404X2S	ICD-10	Poisoning by other synthetic narcotics, intentional self-harm, sequela
T404X3A	ICD-10	Poisoning by other synthetic narcotics, assault, initial encounter
T404X3D	ICD-10	Poisoning by other synthetic narcotics, assault, subsequent encounter
T404X3S	ICD-10	Poisoning by other synthetic narcotics, assault, sequela
T404X4A	ICD-10	Poisoning by other synthetic narcotics, undetermined, initial encounter
T404X4D	ICD-10	Poisoning by other synthetic narcotics, undetermined, subsequent encounter

© 2026 Incze MA et al. *JAMA Network Open*.

T404X4S	ICD-10	Poisoning by other synthetic narcotics, undetermined, sequela
T40601A	ICD-10	Poisoning by unspecified narcotics, accidental (unintentional), initial encounter
T40601D	ICD-10	Poisoning by unspecified narcotics, accidental (unintentional), subsequent encounter
T40601S	ICD-10	Poisoning by unspecified narcotics, accidental (unintentional), sequela
T40602A	ICD-10	Poisoning by unspecified narcotics, intentional self-harm, initial encounter
T40602D	ICD-10	Poisoning by unspecified narcotics, intentional self-harm, subsequent encounter
T40602S	ICD-10	Poisoning by unspecified narcotics, intentional self-harm, sequela
T40603A	ICD-10	Poisoning by unspecified narcotics, assault, initial encounter
T40603D	ICD-10	Poisoning by unspecified narcotics, assault, subsequent encounter
T40603S	ICD-10	Poisoning by unspecified narcotics, assault, sequela
T40604A	ICD-10	Poisoning by unspecified narcotics, undetermined, initial encounter
T40604D	ICD-10	Poisoning by unspecified narcotics, undetermined, subsequent encounter
T40604S	ICD-10	Poisoning by unspecified narcotics, undetermined, sequela
T40691A	ICD-10	Poisoning by other narcotics, accidental (unintentional), initial encounter
T40691D	ICD-10	Poisoning by other narcotics, accidental (unintentional), subsequent encounter
T40691S	ICD-10	Poisoning by other narcotics, accidental (unintentional), sequela
T40692A	ICD-10	Poisoning by other narcotics, intentional self-harm, initial encounter
T40692D	ICD-10	Poisoning by other narcotics, intentional self-harm, subsequent encounter
T40692S	ICD-10	Poisoning by other narcotics, intentional self-harm, sequela
T40693A	ICD-10	Poisoning by other narcotics, assault, initial encounter
T40693D	ICD-10	Poisoning by other narcotics, assault, subsequent encounter
T40693S	ICD-10	Poisoning by other narcotics, assault, sequela
T40694A	ICD-10	Poisoning by other narcotics, undetermined, initial encounter
T40694D	ICD-10	Poisoning by other narcotics, undetermined, subsequent encounter
T40694S	ICD-10	Poisoning by other narcotics, undetermined, sequela

eTable 3. Regression Results

**Total Urine Drug Testing**			
Observations = 64 | F Statistic = 1242.79			
	Coefficient	Standard Error	R Squared
Pre-Intervention	0.67	0.09	<0.001
Post-Intervention	-1.02	0.31	0.002
Change in Slope	-1.69	0.32	<0.001

Dependent Variable = Urine Drug Tests, Independent Variable = Time

© 2026 Incze MA et al. *JAMA Network Open*.

Presumptive Urine Drug Testing			
Observations = 64 | F Statistic = 2036.05			
	Coefficient	Standard Error	R Squared
Pre-Intervention	0.42	0.06	<0.001
Post-Intervention	-0.63	0.14	<0.001
Change in Slope	-1.05	0.15	<0.001

Dependent Variable = Presumptive Urine Drug Test, Independent Variable = Time

Confirmatory Urine Drug Testing			
Observations = 64 | F Statistic = 312.01			
	Coefficient	Standard Error	R Squared
Pre-Intervention	0.25	0.04	<0.001
Post-Intervention	-0.39	0.29	0.18
Change in Slope	-0.65	0.29	0.03

Dependent Variable = Confirmatory Urine Drug Tests, Independent Variable = Time

Overdose Encounters			
Observations = 64 | F Statistic = 37.11			
	Coefficient	Standard Error	R Squared
Pre-Intervention	0.01	0.02	0.47
Post-Intervention	-0.12	0.05	0.01
Change in Slope	-0.14	0.05	0.01

Dependent Variable = Overdose Encounters, Independent Variable = Time

Medication for Opioid Use Disorder Prescriptions			
Observations = 64 | F Statistic = 3397.11			
	Coefficient	Standard Error	R Squared
Pre-Intervention	0.13	0.01	<0.001
Post-Intervention	0.15	0.02	<0.001
Change in Slope	0.01	0.02	0.477

Dependent Variable = Medication for Opioid Use Disorder Prescriptions, Independent Variable = Time

Colonoscopy			
Observations = 64 | F Statistic = 2036.05			
	Coefficient	Standard Error	R Squared

© 2026 Incze MA et al. *JAMA Network Open.*

Pre-Intervention	-0.004	0.01	0.456
Post-Intervention	-0.03	0.02	0.144
Change in Slope	-0.03	0.02	0.204

Dependent Variable = Colonoscopy, Independent Variable = Time

© 2026 Incze MA et al. *JAMA Network Open*.
