# Supplementary material for: Coverage Limitations for Use of Urine Drug Testing in a State Medicaid Program
Source: JAMA Netw Open. 2026 May 8;9(5):e2611711. doi: 10.1001/jamanetworkopen.2026.11711 (PMC13156783; doi:10.1001/jamanetworkopen.2026.11711)
Supplement: Supplement 2. — Data Sharing Statement [file jamanetwopen-e2611711-s002.pdf]

Data Sharing Statement

Incze. Coverage Limitations for Use of Urine Drug Testing in a State Medicaid Program. *JAMA Netw Open*. Published May 08, 2026. doi:10.1001/jamanetworkopen.2026.11711

Data
Data available: No

Additional Information
Explanation for why data not available: Data is property of the Louisiana Department of Public Health
